# Rehabilitation time before disability pension

**DOI:** 10.1186/1472-6963-12-375

**Published:** 2012-10-30

**Authors:** Morten Støver, Kristine Pape, Roar Johnsen, Nils Fleten, Erik R Sund, Bjørgulf Claussen, Solveig Osborg Ose, Johan Håkon Bjørngaard

**Affiliations:** 1 Department of Public Health and General Practice, Faculty of Medicine, Norwegian University of Science and Technology, MTFS, 7491, Trondheim, Norway; 2 Department of Community Medicine, Faculty of Health Science, University of Tromsø, 9037, Tromsø, Norway; 3 Northern Norway Regional Health Authority, Tromsø, 9038 Tromsø, Norway; 4 Department of General Practice and Community Medicine, University of Oslo, 0318, Oslo, Norway; 5 SINTEF Health Research, 7465, Trondheim, Norway; 6 St. Olav's University Hospital Trondheim, Forensic Department and Research Centre Brøset, 7440, Trondheim, Norway

**Keywords:** Disability benefit, Disability pension, Unemployment, Work environment, Multilevel modelling

## Abstract

**Background:**

The decision to grant a disability pension is usually the end of a long process of medical examinations, treatment and rehabilitation attempts. This study investigates to what extent the time spent on rehabilitation time prior to disability pension is associated with characteristics of the individual or the local employment and welfare office, measured as municipality variance.

**Methods:**

A study of 2,533 40 to 42 year olds who received disability pension over a period of 18 years. The logarithm of the rehabilitation time before granting a disability pension was analysed with multilevel regression.

**Results:**

The rehabilitation time before a disability pension was granted ranged from 30 to 5,508 days. Baseline health characteristics were only moderately associated with rehabilitation time. Younger people and people with unemployment periods had longer rehabilitation time before a disability pension was granted. There were only minor differences in rehabilitation time between men and women and between different levels of education. Approximately 2% of the total variance in rehabilitation time could be attributed to the municipality of residence.

**Conclusions:**

There is a higher threshold for granting a disability pension to younger persons and those who are expecting periods of unemployment, which is reflected in the extended rehabilitation requirements for these groups. The longer rehabilitation period for persons with psychiatric disorders might reflect a lack of common knowledge on the working capacity of and the fitted rehabilitation programs for people with psychiatric disorders.

## Background

Disability benefits are important because they provide economical assurance to people who are marginalised from the labour market due to health impairments. The decision to grant a disability pension is in most cases the end of the line of a long process of medical examinations, treatment and rehabilitation attempts. This process is likely to be a substantial strain on the persons involved [1], and the length of the rehabilitation is likely to reflect the anticipated effect of the process, as well as the attitudes and the capacity of the local employment and welfare office.

Although the health of the participant is an important factor when people struggle returning to work after a rehabilitation process, other demographic factors can be important to whether this ends up in employment or receiving a disability pension. Studies have shown that the likelihood of returning to work after rehabilitation decreases with increasing age [2-4] and that individuals with a higher level of education are more likely to return to work [5-7]. The local labour market could also be a deciding factor with respect to work return. Studies have revealed that subjects living in regions with a low level of unemployment were more likely to return to work [8,9], that low national unemployment rates, increases the probability of returning to work [10], and that people living in rural areas were less likely to return to work [11]. A Swedish review [12] presents a number of other demographic factors that are associated with return to work after vocational rehabilitation including working status [2,6], income [13,14] nationality [5,11] and marital status [5,15]. A Swedish study on outcomes of vocational rehabilitation in six local national insurance offices in the same county also revealed major differences in both sickness allowance, return to work and disability pension [16].

In Norway, each municipality has an employment and welfare office that organises social welfare decisions ( http://www.nav.no). Furthermore, each municipality has the responsibility to provide primary health care to its citizens. Although the rules and regulations pertaining to rehabilitation and disability pension are uniform and valid throughout Norway, the legislation on vocational rehabilitation functions as a framework law. As a consequence, each employment and welfare office can exercise discretion in their decisions in the rehabilitation process. This discretion may lead to variations in the rehabilitation process between municipalities, where the employment and welfare offices put more effort in finding and providing more opportunities for rehabilitation for people with better prospects in the labour market, and where disability pensions are given sooner when labour market prospects indicates that a return to work is less likely. Another factor that may differ between municipalities is the quality of the healthcare and the medical rehabilitation for people who have temporarily left the labour market because of health problems.

The aim of this study was to investigate whether there were differences in the duration of the rehabilitation period preceding disability pension between local employment and welfare offices, as measured by municipality variance. The duration of the rehabilitation period between men and women, levels of education, age groups, unemployment status, and diagnoses underlying the disability grant were also investigated.

## Methods

The data were derived from the National Health Screening Service in Norway. Between August 1988 and March 1989 all residents of Nordland County in Norway aged 40 to 42 years were invited to participate. Data were linked to the national insurance database via a personal identification number, created by Statistics Norway and the Norway National Insurance Service. Follow-up time was from January 1^st^ 1992 to December 31^st^ 2007. The Regional Committee for Medical Research Ethics (2009/205-4) approved this study.

Nordland County is situated in the northern region of Norway. At the time of the health screening, Nordland had 45 municipalities and approximately 240,000 inhabitants. Nordland County has a diversity of industries where some municipalities are dominated by fishing, some by agriculture, some by manufacturing industry and some by services. This diversity in industries suggests that municipalities have been affected differently by business fluctuations during the follow-up period.

### Disability pension

Disability pension is granted to people whose earning ability is permanently impaired by at least 50% due to illness, injury or inborn defect. It is also a requirement that the illness or injury is the main reason for the impaired wage earning capacity. Data on new incidents of disability pensions were available from January 1^st^ 1992, and covers all cases of disability pensions in Norway.

### Rehabilitation time before disability pension

The dependent variable in this study was the duration of the rehabilitation period before disability pension. The rehabilitation time in days was calculated as the time between the first date of work disability and the date for granting a disability pension. The first date of work disability represents the point in time when a person’s earning ability was permanently reduced – in most cases the first day of being sick-listed. The time for granted disability pension is always set to three months ahead of the date of application for disability pension. Both dates are registered at the time disability pension is granted. The rehabilitation period normally includes long-term sick leave, medical rehabilitation and vocational rehabilitation programmes which can deal with vocational assessment, work retraining, education, counselling, work guidance and other forms of preparation for returning to work. [13].

### Health measures

In this study, information on different aspects of health and disease were used to adjust for health impairment at baseline. A summarised index of the number of chronic illnesses was constructed including the following conditions: myocardial infarction, angina pectoris, stroke/cerebral infarction, Bechterew’s disease, cancer, diabetes, chronic bronchitis, arthritis, epilepsy, migraine and gastro-intestinal problems. Self-rated health was assessed by the question, “What is your health condition like?” with the four answer categories: “very good,” “good,” “fair” and “poor”. Depression was assessed by the question, “Have you been sad or depressed the last 14 days?” with the four answer categories “almost all the time,” “frequently,” “sometimes” and “never or rarely”. Headache and pains in the neck and shoulders were measured with a four-point scale, with answer categories ranging from “never/rarely” to “daily”. Smoking was assessed with a three-point scale with three answer categories “non-smoker,” “former smoker” and “smoker”. Consumption of alcohol was assessed with a four-point scale, with answer categories ranging from “non-drinker” to “daily drinker.”

### Disability pension diagnosis

Although people can be caused by several diagnoses, the National Work and Welfare Administration codes one major diagnosis after disability pension has been granted. Musculoskeletal and psychiatric diseases are the most common medical diagnoses for being granted a disability pension in Norway [17], and the rehabilitation process could be different for individuals in these diagnostic categories. The study retrieved diagnosis information from the medical classifications ICD-9 and ICD-10. Diagnoses were split into musculoskeletal disorders, psychiatric disorders and “other diagnosis.” To classify individuals in the psychiatric diagnosis group, the ICD-9 mental disorder codes 290–319 and ICD-10 mental disorder codes F00-F99 were used. Individuals with musculoskeletal diagnoses were classified including codes for diseases of the musculoskeletal system and connective tissue 710–739 from ICD-9 and M00-M99 from ICD-10. The diagnosis-specific analysis was restricted to the participants that were registered with a diagnosis at the end of the follow-up (1,346 participants).

### Unemployment

With data obtained from the national insurance register, study participants with any periods of unemployment throughout the follow-up period were classified as having been unemployed.

### Age and education

The age of the participants ranged between 40–42 years at baseline. To investigate whether the duration of the treatment period was different for different age groups; the participants’ ages at the first date of disability was recorded, which ranged from 44 to 61 years. The participants were divided into six age groups. Level of education was measured with the three categories: “primary school”, “high school” and “college/university”.

### Municipality size

A variable was created representing municipality size, reporting whether the respondents were living in a small (less than 7,500 inhabitants), medium (between 7,500 and 15,000 inhabitants) or large municipality (more than 15,000 inhabitants).

### Vocational rehabilitation rates in municipalities

Rates of people on vocational rehabilitation for each municipality for every year of the follow-up ranged from 0.24% to 6.43%. The rehabilitation rate was recorded the same year as the first date of work disability.

### Statistics

The distribution of the rehabilitation time in days was skewed. Accordingly, a log-transformation was performed to correct the skewed data. A linear multilevel regression analysis was applied to individuals nested by municipality of residence and year of start of rehabilitation. To explore the impact of place of residence, the *Intra- class correlation coefficient* (ICC) was calculated as an estimate of the relative importance of place of residence on the length of the rehabilitation period before receiving a disability pension. The main analyses were performed in a three-level model with individuals nested within years within municipality of residence. The diagnosis-specific analyses had no indication of year differences, and thus were performed as a two-level analysis.

The statistical analysis of the duration of the rehabilitation period was performed in three models. Model 1 was adjusted only for age, sex and unemployment. In model 2, baseline health status and health behaviour (as measured by alcohol and smoking behaviour) were added. In model 3, education, municipality size and rehabilitation rate in the municipality were added to model 2’s parameters. The separate analyses for the different diagnoses were done with the same models. The precision of the estimates was presented using 95% confidence intervals (CI). The analyses were limited to the participants with complete information in all study variables (1,757). All analyses were conducted using STATA 11 software (StataCorp LP, Texas, USA).

## Results

### Descriptive results

Of the 10,497 invited to the health screening, 4,302 men and 4,310 women attended, resulting in an attendance rate of 78% and 86% for men and women, respectively [18]. A total of 2,784 (35%) received a disability pension during the follow-up time. Of these respondents 2,533 persons lived in Nordland County at their first date of disability and also were granted disability pension before the end of the follow-up period. A total of 1,757 of the disability pension recipients had complete information on all study variables.

**Figure 1:**
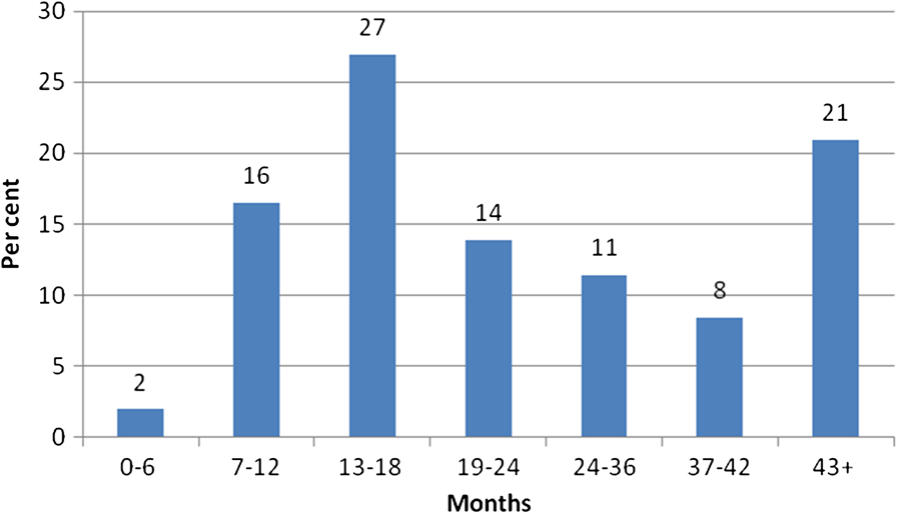
**Distribution of rehabilitation time (%). **N=2,533.

**Figure 2:**
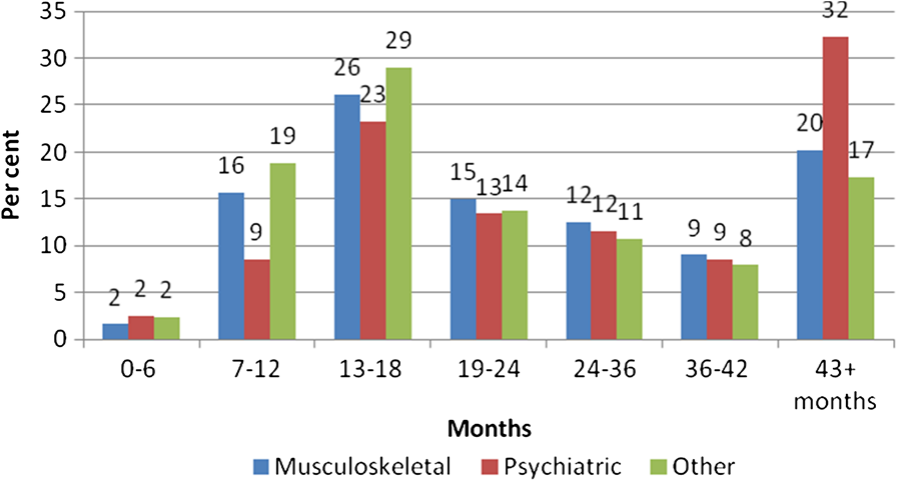
**Distribution of rehabilitation time (%).** Different diagnostic categories underlying the disability pension decision. Musculosceletal (N=689), psychiatric (N=164) and other diagnoses (N=493).

Rehabilitation time for all participants varied from 30 to 5,785 days with a mean of 805 days (2.2 years) and standard deviation of 608 days. In Figure 1, a categorical distribution of rehabilitation time in months is presented. In Figure 2, the same distribution is presented for the different disability diagnostic categories. Those granted a disability pension within the psychiatric diagnosis group had a mean of 847 days (SD 577) rehabilitation time. Those within the musculoskeletal group had a mean of 774 days (SD 518) rehabilitation time, as compared to 751 days (SD 561) for other diagnosis. Table 1 shows rehabilitation time in days for different groups.

**Table 1:** Descriptive statistics. Mean, median and standard deviation of number of days from first day of work disability to day of granted disability pension

	**N**	**Mean**	**Median**	**Std.dev**
Total	2.533	763	579	556
Unemployed in follow-up period	854	875	669	671
Not Unemployed in follow-up period	1679	706	549	477
Chronic illness: 0	1194	759	579.5	537
1	482	748	548.5	591
2 or more	857	775	608	562
Self-rated health: Fair/poor	375	768	550	617
Very good/good	1777	762	580	545
Depressed: Never/rarely/sometimes	1.189	818	579	639
Often/Almost all the time	945	762	579	555
Headache: Never/rarely/Once or several times per month	1837	763	579	763
Once or several times per week/Daily	264	761	607	518
Pain neck/shoulder: Never/rarely/Once or several times per month	1493	748	578	551
Once or several times per week/Daily	589	783	608	562
Smoking: Non-smoker	581	742	578	521
Former smoker	608	744	577	535
Smoker	1343	780	607	579
Alcohol: Non-drinker	838	740	578	533
Up to 1–2 times per month	1012	761	563.5	563
More than once a week/daily	99	856	639	636
Education: Low level	971	773	607	548
Medium level	1287	756	579	563
High level	261	755	577	552
Municipality size: Under 7,500 inhabitants	1055	792	610	592
Between 7,500 and 15,000 inhabitants	615	790	579	590
Over 15,000 inhabitants	863	708	549	477
Musculoskeletal	1002	774	611	518
Psychiatric	261	847	669	577
Diagnosis: “Other”	700	751	563.5	561

^*^Differences in N due to missing data.

Table 2 shows the results from the multilevel linear regression model where the dependent variable was taken as the logarithm of the days of the rehabilitation period before disability pension was granted. The results indicate that there was only minor sex and education differences in the length of the rehabilitation period before disability pension. In the fully adjusted model, the rehabilitation time was approximately 85% shorter for the oldest group than for the youngest (−0.85, 95% CI −0.69 to −1.01). Those experiencing unemployment had a 16% (0.l6, 95% CI 0.10 to 0.22) longer rehabilitation period before they were granted disability pension.

**Table 2:** Multilevel linear regression of the logarithm of days (95% confidence intervals) in rehabilitation time prior to disability pension award

	**Model 1**		**Model 2**		**Model 3**	
	**β**	**95% CI**	**β**	**95% CI**	**β**	**95% CI**
Females vs. Males	0.00	−0.06 to 0.05	0.01	−0.05 to 0.08	0.01	−0.05 to 0.08
Age:						
44-46	Ref		Ref		Ref	
57-59	−0.15	−0.30 to 0.01	−0.17	−0.33 to −0.02	−0.17	−0.33 to −0.02
50-52	−0.28	−0.43 to −0.13	−0.31	−0.47 to −0.16	−0.32	−0.47 to −0.17
53-55	−0.21	−0.36 to −0.06	−0.24	−0.38 to −0.09	−0.26	−0.41 to −0.11
56-58	−0.53	−0.68 to −0.39	−0.56	−0.71 to −0.41	−0.59	−0.75 to −0.44
59-61	−0.80	−0.95 to −0.64	−0.82	−0.98 to −0.67	−0.85	−1.01 to −0.69
Unemployed prior to disability vs. not	0.16	0.10 to 0.22	0.16	0.10 to 0.22	0.16	0.10 to 0.22
Number of reported chronic illnesses			0.03	−0.01 to 0.06	0.03	−0.01 to 0.06
Self-rated health:						
Very good			Ref		Ref	
Good			−0.08	−0.34 to 0.19	−0.07	−0.33 to 0.19
Fair			0.02	−0.23 to 0.28	0.03	−0.23 to 0.29
Poor			0.08	−0.19 to 0.35	0.08	−0.19 to 0.35
Depressed:						
Never/rarely			Ref		Ref	
Sometimes			0.09	−0.15 to 0.34	−0.09	−0.16 to 0.33
Often			0.12	−0.12 to 0.37	0.11	−0.23 to 0.36
Almost all the time			0.14	−0.13 to 0.41	0.13	−0.14 to 0.40
Headache:						
Never/rarely			Ref		Ref	
Once or several times per month			−0.04	−0.10 to 0.03	−0.04	−0.11 to 0.03
Once or several times per week			−0.11	−0.22 to −0.00	−0.11	−0.22 to 0.00
Daily			−0.07	−0.30 to 0.16	−0.07	−0.30 to 0.16
Pain in neck or shoulder:						
Never/rarely			Ref		Ref	
Once or several times per month			0.02	−0.04 to 0.09	0.02	−0.04 to 0.09
Once or several times per week			0.04	−0.06 to 0.14	0.04	−0.06 to 0.14
Daily			0.09	0.00 to 0.18	0.09	0.00 to 0.18
Smoking:						
Non-smoker			Ref		Ref	
Former smoker			−0.00	−0.08 to 0.08	0.00	−0.08 to 0.08
Smoker			−0.02	−0.09 to 0.05	−0.01	−0.09 to 0.06
Alcohol:						
Non-drinker			Ref		Ref	
Up to 1–2 times per month			0.03	−0.03 to 0.10	0.03	−0.03 to 0.10
More than once a week/daily			0.10	−0.03 to 0.24	0.10	−0.03 to 0.24
Education:						
High level					Ref	
Medium level					−0.01	−0.07 to 0.05
Low Level					0.07	−0.03 to 0.16
Municipality size:						
Under 7,500 inhabitants					Ref	
7,500 to 15,000 inhabitants					0.02	−0.07 to 0.11
Over 15,000 inhabitants					−0.07	−0.16 to 0.03
Rehabilitation rate in municipality					0.02	−0.01 to 0.05
Random effects:						
Municipality variance	0.0048		0.0046		0.0041	
Years within municipality variance	0.0026		0.0024		0.0023	
Individual variance	0.3329		0.3268		0.3259	
ICC:	0.02		0.02		0.02	

1,757 individuals in 45 municipalities.

The results in model 1 were based on those having complete information on all study variables. A sensitivity analysis (Additional file 1) of all 2,533 persons who received disability pension gave approximately the same results as those presented in Table 3.

**Table 3:** Multilevel linear regression of the logarithm of days (95% confidence intervals) in rehabilitation time prior to disability pension award for subjects with musculoskeletal diagnosis

	**Model 1**		**Model 2**		**Model 3**	
	**β**	**95% CI**	**β**	**95% CI**	**β**	**95% CI**
Females vs. Males	−0.06	−0.15 to 0.03	−0.05	−0.16 to 0.06	−0.05	−0.16 to 0.06
Age:						
44-46	Ref		Ref		Ref	
47-49	−0.24	−0.45 to −0.02	−0.30	−0.52 to −0.08	−0.29	−0.51 to −0.07
50-52	−0.33	−0.54 to −0.12	−0.40	−0.61 to −0.17	−0.40	−0.60 to −0.18
53-55	−0.38	−0.59 to −0.18	−0.45	−0.66 to −0.24	−0.45	−0.69 to −0.27
56-58	−0.70	−0.92 to −0.50	−0.79	−1.00 to −0.57	−0.79	−1.05 to −0.61
59-61	−0.99	−1.28 to −0.69	−1.05	−1.35 to −0.75	−1.05	−1.40 to −0.79
Unemployed prior to disability vs. not	0.13	0.04 to 0.23	0.14	0.05 to 0.24	0.14	0.04 to 0.23
Number of reported chronic illnesses			0.01	−0.04 to 0.06	0.01	−0.04 to 0.06
Self-rated health:						
Very good			Ref		Ref	
Good			−0.09	−0.49 to 0.32	−0.06	−0.46 to 0.34
Fair			0.11	−0.29 to 0.50	0.13	−0.27 to 0.53
Poor			0.23	−0.18 to 0.65	0.25	−0.16 to 0.67
Depressed:						
Never/rarely			Ref		Ref	
Sometimes			−0.09	−0.47 to 0.29	−0.08	−0.46 to 0.30
Often			−0.05	−0.44 to 0.33	−0.05	−0.43 to 0.33
Almost all the time			−0.05	−0.47 to 0.37	−0.05	−0.44 to 0.40
Headache:						
Never/rarely			Ref		Ref	
Once or several times per month			−0.03	−0.13 to 0.07	−0.03	−0.13 to 0.07
Once or several times per week			−0.06	−0.22 to 0.10	−0.06	−0.22 to 0.10
Daily			0.04	−0.33 to 0.40	0.05	−0.31 to 0.42
Pain in neck or shoulder:						
Never/rarely			Ref		Ref	
Once or several times per month			0.01	−0.14 to 0.12	0.00	−0.11 to 0.12
Once or several times per week			0.07	−0.08 to 0.22	0.07	−0.08 to 0.22
Daily			0.13	−0.02 to 0.27	0.12	−0.02 to 0.26
Smoking:						
Non-smoker			Ref		Ref	
Former smoker			−0.03	−0.15 to 0.10	−0.03	−0.16 to 0.10
Smoker			−0.02	−0.14 to 0.09	−0.03	−0.15 to 0.08
Alcohol:						
Non-drinker			Ref		Ref	
Up to 1–2 times per month			0.04	−0.07 to 0.14	0.04	−0.06 to 0.14
More than once a week/daily			0.10	−0.13 to 0.33	0.11	−0.12 to 0.34
Education:						
High level					Ref	
Medium level					−0.03	−0.12 to 0.06
Low Level					0.01	−0.17 to 0.20
Municipality size:						
Under 7,500 inhabitants					Ref	
7,500 to 15,000 inhabitants					−0.06	−0.19 to 0.08
Over 15,000 inhabitants					−0.09	−0.24 to 0.05
Rehabilitation rate in municipality					0.04	−0.01 to 0.09
Random effects:						
Variance between municipalities	0.0076		0.0072		0.0077	
Variance within municipalities	0.3266		0.3175		0.3153	
ICC:	0.02		0.02		0.02	

689 individuals in 45 municipalities.

### Municipality differences in rehabilitation time

The multilevel analysis indicated relatively small differences between the practices of the employment and welfare offices in the length of rehabilitation periods. The ICC at the municipality level was between 1 and 2% in all models in Table 2. However, the ICC was statistically significant (p<.01 in all three models), suggesting that the municipality differences were greater than what would be expected due to chance alone.

### Diagnosis specific analyses

Analyses for the different groups of disability diagnosis are presented in Tables 3, 4 and 5. For people with “other” diagnosis and those in the musculoskeletal group, the ICC was between 1 and 2% in all models. For the psychiatric group, model 1 gives an ICC of 17%. Adjusting for health, smoking and alcohol use reduced the ICC to 12% and in model 3 the ICC was reduced to zero. Several models were performed to determine the robustness of the crude high ICC for psychiatric diagnoses. The number of individuals with complete survey information and a psychiatric disability diagnosis was low (n=164). A sensitivity analysis (Additional file 1) of all 261persons who received disability pension with a psychiatric diagnosis gave an ICC of about 1%, suggesting an ICC in line with the other models of our analyses.

**Table 4:** Multilevel linear regression of the logarithm of days (95% confidence intervals) in rehabilitation time prior to disability pension award for subjects with psychiatric diagnosis

	**Model 1**		**Model 2**		**Model 3**	
	**β**	**95% CI**	**β**	**95% CI**	**β**	**95% CI**
Females vs. Males	0.10	−0.10 to 0.30	0.08	−0.15 to 0.32	0.15	−0.08 to 0.37
Age:						
44-46	Ref		Ref		Ref	
47-49	−0.13	−0.56 to 0.31	−0.05	−0.24 to 0.22	−0.02	−0.44 to 0.41
50-52	−0.10	−0.53 to 0.33	−0.08	−0.69 to −0.05	−0.03	0.46 to 0.39
53-55	0.01	−0.42 to 0.44	0.04	−0.39 to 0.47	0.04	−0.39 to 0.48
56-58	−0.40	−0.85 to 0.06	−0.38	−0.84 to 0.08	−0.32	−0.80 to 0.16
59-61	-	-	-	-	-	-
Unemployed prior to disability vs. not	0.09	−0.14 to 0.31	0.05	−0.18 to 0.27	−0.01	−0.23 to 0.21
Number of reported chronic illnesses			−0.01	−0.12 to 0.10	−0.02	−0.12 to 0.09
Self-rated health:						
Very good			Ref		Ref	
Good			−0.63	−1.63 to 0.37	−0.60	−1.58 to 0.39
Fair			−0.52	−1.51 to 0.46	−0.47	−1.43 to 0.49
Poor			0.47	−1.52 to 0.57	−0.42	−1.45 to 0.61
Depressed:						
Never/rarely			Ref		Ref	
Sometimes			−0.19	−0.79 to 0.41	−0.29	−0.87 to 0.28
Often			−0.20	−0.79 to 0.39	−0.33	−0.90 to 0.23
Almost all the time			−0.28	−0.94 to 0.38	−0.43	−1.06 to 0.21
Headache:						
Never/rarely			Ref		Ref	
Once or several times per month			−0.17	−0.41 to 0.08	−0.17	−0.42 to 0.08
Once or several times per week			−0.58	−0.96 to 0.20	−0.70	−1.07 to 0.34
Daily			−0.54	−1.22 to 0.14	−0.46	−1.13 to 0.22
Pain in neck or shoulder:						
Never/rarely			Ref		Ref	
Once or several times per month			0.09	−0.17 to 0.35	0.14	−0.11 to 0.40
Once or several times per week			0.41	0.02 to 0.80	0.50	0.12 to 0.89
Daily			0.36	0.03 to 0.70	0.45	0.12 to 0.78
Smoking:						
Non-smoker			Ref		Ref	
Former smoker			−0.21	−0.53 to 0.11	−0.18	−0.49 to 0.13
Smoker			−0.15	−0.44 to 0.13	0.08	−0.36 to 0.20
Alcohol:						
Non-drinker			Ref		Ref	
Up to 1–2 times per month			0.12	−0.12 to 0.36	0.18	−0.06 to 0.42
More than once a week/daily			−0.00	−0.41 to 0.40	0.06	−0.34 to 0.45
Education:						
High level					Ref	
Medium level					0.06	−0.16 to 0.29
Low Level					0.40	0.12 to 0.69
Municipality size:						
Under 7,500 inhabitants					Ref	
7,500 to 15,000 inhabitants					0.02	−0.23 to 0.28
Over 15,000 inhabitants					−0.35	−0.57 to −0.12
Rehabilitation rate in municipality					−0.05	−0.14 to 0.08
Random effects:						
Variance between municipalities	0.0756		0.0477		0.0000	
Variance within municipalities	0.3706		0.3513		0.3599	
ICC:	0.17		0.12		0.00	

164 individuals in 45 municipalities.

**Table 5:** Multilevel linear regression of the logarithm of days (95% confidence intervals) in rehabilitation time prior to disability pension award for subjects with other diagnoses

	**Model 1**		**Model 2**		**Model 3**	
	**β**	**95% CI**	**β**	**95% CI**	**β**	**95% CI**
Females vs. Males	−0.02	−0.11 to 0.08	0.04	−0.08 to 0.15	0.03	−0.09 to 0.15
Age:						
44-46	Ref		Ref		Ref	
47-49	0.02	−0.25 to 0.30	−0.25	−0.25 to −0.30	0.03	−0.25 to 0.31
50-52	−0.26	−0.52 to 0.01	−0.26	−0.54 to 0.01	−0.26	−0.53 to 0.02
53-55	−0.14	−0.40 to 0.13	−0.12	−0.40 to 0.15	−0.10	−0.39 to 0.17
56-59	−0.52	−0.79 to −0.26	−0.50	−0.78 to −0.23	−0.48	−0.76 to −0.20
60-62	−0.80	−1.17 to −0.44	−0.77	−1.14 to −0.40	−0.74	−1.13 to −0.35
Unemployed prior to disability vs. not	0.15	0.05 to 0.25	0.19	0.08 to 0.29	0.19	0.08 to 0.30
Number of reported chronic illnesses			0.05	−0.00 to 0.10	−0.05	−0.01 to 0.10
Self-rated health:						
Very good			Ref		Ref	
Good			−0.01	−0.43 to 0.42	−0.02	−0.44 to 0.41
Fair			0.11	−0.30 to 0.53	0.11	−0.31 to 0.53
Poor			0.09	−0.35 to 0.53	0.09	−0.36 to 0.53
Depressed:						
Never/rarely			Ref		Ref	
Sometimes			0.01	−0.57 to 0.58	0.00	−0.57 to 0.58
Often			0.04	−0.54 to 0.62	0.04	−0.54 to 0.61
Almost all the time			−0.04	−0.66 to 0.57	−0.05	−0.67 to 0.57
Headache:						
Never/rarely			Ref		Ref	
Once or several times per month			−0.11	−0.24 to 0.01	−0.12	−0.25 to 0.01
Once or several times per week			−0.09	−0.30 to 0.12	−0.10	−0.31 to 0.11
Daily			0.01	−0.42 to 0.44	0.01	−0.42 to 0.44
Pain in neck or shoulder:						
Never/rarely			Ref		Ref	
Once or several times per month			0.09	−0.04 to 0.21	0.09	−0.04 to 0.22
Once or several times per week			0.10	−0.08 to 0.29	0.11	−0.08 to 0.30
Daily			0.13	−0.05 to 0.32	0.14	−0.04 to 0.32
Smoking:						
Non-smoker			Ref		Ref	
Former smoker			−0.04	−0.19 to 0.11	−0.03	−0.19 to 0.12
Smoker			−0.01	−0.14 to 0.12	−0.01	−0.14 to 0.12
Alcohol:						
Non-drinker			Ref		Ref	
Up to 1–2 times per month			0.06	−0.07 to 0.18	0.05	−0.07 to 0.18
More than once a week/daily			0.24	−0.02 to 0.49	0.25	−0.01 to 0.51
Education:						
High level					Ref	
Medium level					0.03	−0.09 to 0.14
Low Level					0.04	−0.13 to 0.21
Municipality size:						
Under 7,500 inhabitants					Ref	
7,500 to 15000 inhabitants					−0.06	−0.20 to 0.07
Over 15,000 inhabitants					−0.02	−0.14 to 0.10
Rehabilitation rate in municipality					−0.02	−0.07 to 0.03
Random effects:						
Variance between municipalities	0.0038		0.0025		0.0008	
Variance within municipalities	0.3136		0.3048		0.3053	
ICC:	0.01		0.01		0.00	

493 individuals in 45 municipalities.

## Discussion

### Main findings

The results from this large population study showed considerable variation in the time before a disability pension are granted, ranging from 30 to 5,508 days. As expected, younger age was associated with a longer rehabilitation time. However, the initial health of the study participants was only marginally associated with the time of the rehabilitation period. Furthermore, those who experienced unemployment periods in the follow up period had longer rehabilitation time before a disability pension was granted than those not being unemployed. There were only minor differences in rehabilitation time before disability pension for men or women, or for different levels of education. Approximately 2% of the total variance could be attributed to the municipality level. The municipality rate of vocational rehabilitation had no substantial influence on rehabilitation time.

### Strengths and limitations

The present study was a large population based survey with a high response rate (82%). The information in this study was obtained from a highly reliable source established by Statistics Norway and the Norway Social Insurance Service. Although numerous studies are published on rehabilitation and return to work, this is, to our knowledge, the first study that investigates variations in the duration of the rehabilitation period for a group of participants ultimately becoming disability pension recipients.

The accuracy of the rehabilitation time period is presumably high as the information was obtained from a highly reliable source set up by Statistics Norway and the Norway Social Insurance Service.

The questionnaire in this study did not contain formerly validated health scales. However, the study had comprehensive information on several diseases and complaints that are well known risk factors for disability pension. Furthermore, the study included self-rated health, a common measure for both physical and mental health and also an independent predictor for disability pension [19-21]. The present study had only a crude measure of alcohol consumption, which may have underestimated the impact of alcohol consumption.

The number of participants was limited to those with complete information for all study variables (1,757) in the regression models. There might be selection effects in the study, meaning that the respondents who chose not to answer questions about their health or health behaviour may have experienced a different rehabilitation pattern and rehabilitation time than those included. The diagnosis-specific analysis was limited to the participants that were registered with a diagnosis at time end of the follow-up (1,346). The diagnosis for disability pension can be delayed for some persons, meaning that our data had missing information about diagnosis for some of the participants that received disability pension the last years of the follow-up.

This study considered rehabilitation time only for those who eventually were granted disability pension, and the results of the rehabilitation process may have differed if we had included those succeeding return to work.

The study did not have full information on disability pension and unemployment from 1990 and 1991. Hence, information from the participants starting their disability process before 1992 was not available.

### Rehabilitation time before disability pension

Age was associated with the length of the rehabilitation period. Several other studies has shown that the chances of job return after a rehabilitation period is attenuated with increasing age [3,22]. This attenuation may be because job return seems to be more likely for younger people who have a better overall health and who are more attractive on the labour market. Younger people who are granted a disability pension lose more productive years, and it is likely that the employment and welfare offices are more prone to facilitating job return for younger people, hence a longer and more thorough rehabilitation process before granting a disability pension.

The length of the rehabilitation process was approximately the same for different levels of education. Although a recent Norwegian study [22] concluded that educational level had no substantial influence on the probability of returning to work after rehabilitation, most previous studies have shown that people with higher education are more likely to succeed returning to work after rehabilitation [5-7]. One might expect that highly educated persons have more opportunities in terms of finding new jobs. This study considered rehabilitation time only for those who eventually were granted disability pension, and if we studied the results of the rehabilitation process the findings may have differed. A reason could be that higher educated individuals who apply for a disability pension have more disabling conditions than lower educated individuals. The analysis did not indicate any substantial differences between men and women regarding the length of rehabilitation before the granting of the disability pension. Previous research has shown conflicting findings in terms of sex differences in the likelihood of returning to work. A Swedish review [12] showed that even though a majority of the studies indicate that men are more successful in returning to work after a rehabilitation period, others indicate the opposite. Again, this study could not answer whether there are sex differences in results of a rehabilitation process, only whether there are differences in duration of the rehabilitation process between the sexes.

People who experienced unemployment in the follow-up period had a longer rehabilitation period before disability pension was granted. Previous studies have shown that having a job to return to is associated with returning to work after a rehabilitation period, compared with those without a job to return to [2,6]. A longer rehabilitation period for people who have been unemployed could be caused by difficulties in assessing the major cause of their work incapacity, their health impairments or their unemployment situation.

One would expect poor health to be associated with a shorter rehabilitation period, given that poor health is a premise for being granted a disability pension. However, in this study health measures were only marginally associated with the length of the rehabilitation period. Several studies have shown that people with more severe diseases are less likely to return to work [14,15], and it is important to notice that this study had information on baseline health only; no information was collected on health throughout the follow up period. It is also possible that the sample heterogeneity was reduced, for education and gender differences, because only those that were granted a disability pension were studied.

### Municipality differences

The multilevel analysis showed that 2% of the variance could be attributed the municipality level. These results might indicate fairly equal practice between social service offices across municipalities. This is also in line with the results of a previous study based on the same material, assessing the risk of disability pension between the different municipalities where approximately 2% of the variance could be attributed to the municipality level [23].

Previous studies have shown that subjects living in regions with a low level of unemployment were more likely to return to work [8,9], and that people living in the countryside were less likely to return to work [11]. Although health is the most important factor for succeeding returning to work, work place characteristics could also be of importance. For people with manual work, or with few opportunities for adjustments at their original workplace, health impairments can make it more difficult returning to work, compared to those who have the possibility to adapt to other tasks. This means that area of residence can be of more importance for some people, especially for those who have problems returning to their original workplace, and have to search for jobs in areas with high unemployment rates, or in rural areas with less employment opportunities.

The present study’s results indicated that people with psychiatric diagnoses were granted a disability pension sooner in the largest municipalities. This finding may be due to organisational characteristics or other characteristics of some employment and welfare offices in some large municipalities. Hence, this finding requires more research attention. One interpretation of this finding is that the employment and welfare offices in the smallest municipalities have less experience with people with psychiatric diagnoses, have more problems assessing their work capacity and has a lack of knowledge on suitable rehabilitation programmes for this diagnostic group.

## Conclusions

This study revealed a longer rehabilitation time for younger people and those who have experienced unemployment during the follow-up period. Higher thresholds for granting a disability pension to younger persons and for those having experienced unemployment can reflect a demand for extended rehabilitation measures for these groups. Baseline health characteristics were only moderately associated with rehabilitation time, and no substantial differences in rehabilitation time between men and women, or for different levels of education were found This result may be explained by the fact that the heterogeneity among employees is strongly reduced when we study only those that are granted disability pension. This sample is thus adjusted for all factors that affect the probability of being granted a disability pension (health, gender, education etc.). Place of residence had modest importance for the length of the rehabilitation time. Larger municipalities had a considerably shorter rehabilitation time before the granting of a disability pension. The longer rehabilitation period for persons with psychiatric disorders could reflect difficulties assessing their working capacity and a lack of knowledge on rehabilitation programs for this group.

## Competing interests

The authors declare that they have no competing interests.

## Authors’ contributions

MS carried out the data processing, the epidemiological modeling and statistical analysis and wrote the manuscript. KP, RJ and JHB contributed to the epidemiological modeling, statistical analysis, data interpretation and drafting of the manuscript. NF, ES, SOS and BC participated in the design of the study and helped to write the manuscript. All authors read and approved the final manuscript.

## Pre-publication history

The pre-publication history for this paper can be accessed here:

http://www.biomedcentral.com/1472-6963/12/375/prepub

## Supplementary Material

Additional file 1 **Appendix: Table 6.** Multilevel linear regression of the logarithm of days (95% confidence intervals) in rehabilitation time prior to disability pension award. Complete case. (DOC 44 kb) Click here for file

## References

[B1] Overland S Glozier N Henderson M Maeland JG Hotopf M Mykletun A Health status before, during and after disability pension award: the Hordaland Health Study (HUSK) Occup Environ Med 2008 65 11 769 773 10.1136/oem.2007.037861 18940958

[B2] Marnetoft SU Selander J Bergroth A Ekholm J Factors associated with successful vocational rehabilitation in a Swedish rural area J Rehabil Med 2001 33 2 71 78 10.1080/165019701750098902 11474952

[B3] Selander J Marnetoft SU Asell M Predictors for successful vocational rehabilitation for clients with back pain problems Disabil Rehabil 2007 29 3 215 220 10.1080/09638280600756208 17364772

[B4] Crook J Moldofsky H Shannon H Determinants of disability after a work related musculetal injury J Rheumatol 1998 25 8 1570 1577 9712103

[B5] Hennessey JC Muller LS The effect of vocational rehabilitation and work incentives on helping the disabled-worker beneficiary back to work Soc Secur Bull 1995 58 1 15 28 7644967

[B6] Voaklander D Beaulne A RA L Factors related to outcome following a work hardening program Journal of Occpational Rehabilitation 1995 5 2 71 85 10.1007/BF02109911 24234578

[B7] Straaton KV Maisiak R Wrigley JM Fine PR Musculoskeletal disability, employment, and rehabilitation J Rheumatol 1995 22 3 505 513 7783071

[B8] Cook J Mulkern V Grey D Burke-Miller J Blyler C Razzano L Onken S Balser R Gold P Shafer M Effects of local unemployment rate on vocational outcomes in a randomized trial of supported employment for individuals with psychiatric disabilities Journal of Vocational Rehabilitation 2006 25 71 84

[B9] Sheikh K Mattingly S Employment Rehabilitation: Outcome and Prediction Am J Ind Med 1984 5 383 393 10.1002/ajim.4700050507 6232844

[B10] Misra S Tseng m Influence of the Unemployment Rate on Vocational Rehabilitation Closures Rehabilitation Counselling Bulletin 1986 29 3 158 165

[B11] Heikkila H Heikkila E Eisemann M Predictive factors for the outcome of a multidisciplinary pain rehabilitation programme on sick-leave and life satisfaction in patients with whiplash trauma and other myofascial pain: a follow-up study Clin Rehabil 1998 12 6 487 496 10.1191/026921598670569564 9869252

[B12] Selander J Marnetoft SU Bergroth A Ekholm J Return to work following vocational rehabilitation for neck, back and shoulder problems: risk factors reviewed Disabil Rehabil 2002 24 14 704 712 10.1080/09638280210124284 12396655

[B13] Selander J Unemployed sick leavers and vocational rehabilitation 1999 A person level study based on a national social insurance material. Dissertation from the Department of Rehabilitation, Karonlinska Institute

[B14] Tate DG Workers' disability and return to work Am J Phys Med Rehabil 1992 71 2 92 96 10.1097/00002060-199204000-00006 1532720

[B15] Jang Y Li W Hwang M WY C Factors Related to Returning to Work Following a Work-Oriented Occupational Therapy Program for Individuals with Physical Disabilities J Occup Rehabil 1998 8 2 141 151 10.1023/A:1023067707057

[B16] Ahlgren A Broman L Bergroth A Ekholm J Disability pension despite vocational rehabilitation? A study from six social insurance offices of a county Int J Rehabil Res 2005 28 1 33 42 10.1097/00004356-200503000-00005 15729095

[B17] Mykletun A Knudsen AK Lost years of working due to disability pension award for mental disorders 2009 Norwegian Institute of Public Health, Oslo

[B18] Jacobsen BK Stensvold I Fylkesnes K Kristiansen IS Thelle DS The Nordland Health Study. Design of the study, description of the population, attendance and questionnaire response Scand J Soc Med 1992 20 3 184 187 1485156 10.1177/140349489202000309

[B19] Krokstad S Johnsen R Westin S Social determinants of disability pension: a 10-year follow-up of 62 000 people in a Norwegian county population Int J Epidemiol 2002 31 6 1183 1191 10.1093/ije/31.6.1183 12540720

[B20] Virtanen M Kivimäki M Singh-Manoux A Gimeno D Shipley M Vahtera J Akbaraly T Marmot M Ferrie J Work disability following major organisational change: the Whitehall II study J Epidemiol Community Health 2010 64 5 4 10.1136/jech.2009.095158 PMC2997797 20445214

[B21] Månsson N Råstam L Self-rated health as a predictor of disability pension and death - A prospective study of middle-aged men Scand J Public Health 2001 29 151 158 11484868

[B22] Landstad BJ Wendelborg C Hedlund M Factors explaining return to work for long-term sick workers in Norway Disabil Rehabil 2009 31 15 1215 1226 10.1080/09638280802510999 19280436

[B23] Stover M Pape K Johnsen R Fleten N Sund ER Claussen B Bjorngaard JH Unemployment and disability pension-an 18-year follow-up study of a 40-year-old population in a Norwegian county BMC Publ Health 2012 12 148 10.1186/1471-2458-12-148 PMC3305666 22369630

