# Supplementary material for: Rehabilitation time before disability pension
Source: BMC Health Serv Res. 2012 Oct 30;12:375. doi: 10.1186/1472-6963-12-375 (PMC3504554; doi:10.1186/1472-6963-12-375)
Supplement: Additional file 1 — Appendix: Table 6. Multilevel linear regression of the logarithm of days (95% confidence intervals) in rehabilitation time prior to disability pension award. Complete case. (DOC 44 kb) [file 1472-6963-12-375-S1.doc]

Appendix: Table 6: Multilevel linear regression of the logarithm of days (95% confidence intervals) in rehabilitation time prior to disability pension award. Complete case

|  | Model 1^1^ | |  | Model 1^2^ | |  | Model 1^3^ | |  | Model 1^4^ | |
| --- | --- | --- | --- | --- | --- | --- | --- | --- | --- | --- | --- |
|  | β | 95% CI |  | β | 95% CI |  | β | 95% CI |  | β | 95% CI |
|  |  |  |  |  |  |  |  |  |  |  |  |
| Females vs. Males | 0.01 | -0.03 to 0.06 |  | -0.02 | -0.10 to 0.06 |  | 0.06 | -0.10 to 0.22 |  | 0.02 | -0.07 to 0.10 |
| Age: |  |  |  |  |  |  |  |  |  |  |  |
| 44-46 | Ref |  |  | Ref |  |  | Ref |  |  | Ref | Ref |
| 47-49 | -0.21 | -0.35 to -0.08 |  | -0.20 | -0.39 to 0.01 |  | -0.32 | -0.64 to 0.01 |  | -0.17 | -0.39 to 0.06 |
| 50-52 | -0.30 | -0.42 to -0.17 |  | -0.33 | -0.51 to -0.14 |  | -0.21 | -0.54 to 0.12 |  | -0.35 | -0.57 to -0.13 |
| 53-55 | -0.25 | -0.37 to -0.12 |  | -0.32 | -0.51 to -0.14 |  | 0.12 | -0.45 to 0.21 |  | -0.33 | -0.55 to -0.13 |
| 56-58 | -0.54 | -0.67 to- 0.41 |  | -0.66 | -0.85 to -0.48 |  | -0.52 | -0.87 to -0.17 |  | -0.63 | -0.85 to -0.41 |
| 59-61 | -0.82 | -0.95 to -0.68 |  | -0.98 | -1.24 to -0.72 |  | -0.37 | -1.65 to 0.92 |  | -1.02 | -1.31 to -0.73 |
| Unemployed prior to disability vs. not | 0.16 | 0.12 to 0.21 |  | 0.13 | 0.06 to 0.21 |  | 0.14 | -0.04 to 0.32 |  | 0.20 | 0.11 to 0.29 |
|  |  |  |  |  |  |  |  |  |  |  |  |
| Random effects: |  |  |  |  |  |  |  |  |  |  |  |
| Variance between municipalities | 0.0062 |  |  | 0.0060 |  |  | 0.0027 |  |  |  | 0.0103 |
| Variance within municipalities | 0.3284 |  |  | 0.3246 |  |  | 0.4088 |  |  |  | 0.3119 |
| ICC: | 0.02 |  |  | 0.02 |  |  | 0.01 |  |  |  | 0.03 |

^1^ Model 1 from table 1. n=2,533

^2^ Model 1 from table 2. Musculoskeletal diagnosis: n=1,002

^3^ Model 1 from table 3. Psychiatric diagnosis: n=261

^4^ Model 1 from table 4. Other diagnosis n=700
